# Post–COVID-19 Condition Fatigue Outcomes Among Danish Residents

**DOI:** 10.1001/jamanetworkopen.2024.34863

**Published:** 2024-10-07

**Authors:** Elisabeth O’Regan, Lampros Spiliopoulos, Ingrid Bech Svalgaard, Nete Munk Nielsen, Anna Irene Vedel Sørensen, Peter Bager, Poul Videbech, Steen Ethelberg, Anders Koch, Anders Hviid

**Affiliations:** 1 Department of Epidemiology Research, Statens Serum Institut, Copenhagen, Denmark; 2 Focused Research Unit in Neurology, Department of Neurology, Hospital of Southern Jutland, University of Southern Denmark, Aabenraa, Denmark; 3 Infectious Disease Epidemiology and Prevention, Statens Serum Institut, Copenhagen, Denmark; 4 Center for Neuropsychiatric Depression Research, Mental Health Center Glostrup, Glostrup, Denmark; 5 Clinical Institute, University of Copenhagen, Copenhagen, Denmark; 6 Department of Public Health, Global Health Section, University of Copenhagen, Copenhagen, Denmark; 7 Department of Infectious Diseases, Rigshospitalet University Hospital, Copenhagen, Denmark; 8 Pharmacovigilance Research Center, Department of Drug Design and Pharmacology, University of Copenhagen, Copenhagen, Denmark

## Abstract

**Importance:**

Fatigue remains one of the most common and debilitating symptoms of post–COVID-19 condition; however, existing studies are limited to select populations and often lack noninfected controls. It also remains unclear to what extent severity of infection and psychiatric conditions, which are often linked to chronic fatigue, modify the risk of post–COVID-19 condition fatigue symptoms.

**Objective:**

To evaluate the impact of SARS-CoV-2 infection on self-reported fatigue and postexertional malaise over time and to explore possible risk factors, such as the impact of acute SARS-CoV-2 hospitalization and preexisting psychiatric conditions on postacute fatigue.

**Design, Setting, and Participants:**

In this cohort study, Danish residents aged 15 years and older were invited to participate in the EFTER-COVID survey, which used repeated, self-reported online questionnaires that collected information on fatigue (Fatigue Assessment Scale) and postexertional malaise scores (DePaul Symptom Questionnaire) after individuals’ index SARS-CoV-2 polymerase chain reaction test. Participants were included if they completed a baseline and at least 1 follow-up questionnaire 2 to 18 months after testing for SARS-CoV-2.

**Exposure:**

Testing for SARS-CoV-2 infection.

**Main Outcomes and Measures:**

The primary outcomes were fatigue and postexertional malaise 2 to 18 months after testing. Mixed-effects models were used to compare scores between SARS-CoV-2 test-positive and test-negative individuals (testing period April 2021 to February 2023).

**Results:**

Of a total of 50 115 participants (median [IQR] age at test date, 57 [46-67] years; 29 774 female [59.4%]), 25 249 were test positive and 24 866 were test negative. Most participants were vaccinated with at least 2 doses (21 164 test-negative participants [85.1%] and 22 120 test-positive participants [87.6%]) before their SARS-CoV-2 index test and fatigue reporting. In the period 2 to 18 months after testing, SARS-CoV-2 infection was associated with a small but significant 3% increase in self-reported fatigue scores (score ratio [SR], 1.03; 95% CI, 1.03-1.04) and higher odds of self-reported postexertional malaise (odds ratio, 2.04; 95% CI, 1.81-2.30), compared with test-negative participants. In the same period, hospitalization with SARS-CoV-2 increased fatigue scores by 23% (SR, 1.23; 95% CI, 1.20-1.26) compared with test-negative participants. Preexisting psychiatric conditions did not significantly modify postacute fatigue scores.

**Conclusions and Relevance:**

In this cohort study, SARS-CoV-2 infection was associated with a subtle increase in self-reported fatigue and postexertional malaise symptoms 2 to 18 months after mild infection. In contrast, individuals hospitalized with acute SARS-CoV-2 experienced a more substantial increase in postacute symptoms. Preexisting psychiatric conditions did not significantly modify the risk of postacute fatigue symptoms. The findings largely captured symptoms following first-time infections in a population where most had been vaccinated. Persons who experienced severe acute infection may benefit from clinical follow-up for fatigue.

## Key Points

**Question:**

Is SARS-CoV-2 infection associated with self-reported fatigue and postexertional malaise over time?

**Findings:**

In this population-based cohort study of 50 115 Danish participants, where most of the study population was vaccinated before testing for SARS-CoV-2, a 3% increase in self-reported fatigue and 2-fold increase in symptoms of postexertional malaise were found 2 to 18 months after infection, compared with noninfected controls. In the same period, persons hospitalized with acute SARS-CoV-2 infection experienced a 23% increase in fatigue.

**Meaning:**

The burden of post–COVID-19 condition fatigue was highest among patients with more severe cases of infection and was long-lasting, suggesting that patients with severe acute infection may benefit from clinical follow-up for fatigue.

## Teaser

This cohort study evaluates the association of SARS-CoV-2 infection with self-reported fatigue and postexertional malaise over time.

## Introduction

Post–COVID-19 condition (PCC) often resembles myalgic encephalomyelitis and chronic fatigue syndrome,^1,2^ which can be disruptive to quality of life and debilitating.^3^ Worsening exhaustion following physical or mental activity, termed postexertional malaise (PEM), has also been considered a hallmark of PCC.^4^ Prevalence estimates have shown that approximately 1 in 3 individuals experience fatigue lasting 3 months or more after their initial infection.^5,6^ However, the incidence of chronic fatigue after SARS-CoV-2 infection is not well understood, as many studies on the subject lack never-infected control groups or are limited to select study populations, such as health care staff, veterans, or persons hospitalized with acute SARS-CoV-2.

Several studies have pointed to female sex, middle to older age, high body mass index (BMI; calculated as weight in kilograms divided by height in meters squared), and hospitalization with acute SARS-CoV-2 as risk factors for PCC.^7^ Some studies have also observed an increased risk of PCC among individuals with psychiatric conditions, including anxiety and depression,^7,8,9,10^ and these populations are often predisposed to symptoms of chronic fatigue, tiredness, or low energy.^11,12^ However, other findings have disputed associations between psychiatric conditions and PCC development.^13,14^

Although some researchers have examined register-based outcomes to identify possible PCC cases,^13^ many individuals experiencing chronic fatigue may not seek help from health care settings and receive a diagnosis. For this reason, we examined repeated, validated, survey-based measures of self-reported fatigue in a Danish subpopulation and compared outcomes between persons infected with SARS-CoV-2 and persons with no known history of SARS-CoV-2. We, therefore, aimed to (1) evaluate the association of SARS-CoV-2 infection with self-reported fatigue and PEM over time and (2) explore the association of acute SARS-CoV-2 hospitalization, preexisting psychiatric conditions, and other possible risk factors with postacute fatigue.

## Methods

### Study Design and Context

In this population-based cohort study, nationwide survey and register data were linked using the unique identifier (the CPR number) assigned to all Danish residents in the Danish Civil Registration System. According to Danish law, national surveillance activities performed by Statens Serum Institut do not require approval from an ethics committee. This report follows Strengthening the Reporting of Observational Studies in Epidemiology (STROBE) reporting guidelines for cohort studies.

In Denmark, widespread reverse-transcription polymerase chain reaction testing for SARS-CoV-2 began at the end of May 2020 and continued until March 2022, when all test results were registered in the Danish microbiology database (MiBa). Periods of variant dominance were informed by results from extensive national whole-genome sequencing.^15,16^

### The EFTER-COVID Survey

The EFTER-COVID (AFTER COVID) survey is a nationwide survey designed to monitor PCC symptoms in the Danish population.^17^ Invitations were initiated by MiBa-registered reverse-transcription polymerase chain reaction test results and administered using Denmark’s mandatory digital mail system, e-Boks. We examined individuals who contributed their index test (the test initiating an invitation to participate in the survey) for SARS-CoV-2 as early as April 3, 2021, and as late as February 21, 2023. Follow-up questionnaires, contingent on participant consent, were sent out 2, 4, 6, 9, 12, and 18 months after each individual’s index test date (eFigure 1 in Supplement 1).

Here, we examine responses from the fatigue track of the survey, which was based on the Fatigue Assessment Scale (FAS)^18^ and PEM questions from the DePaul Symptom Questionnaire. Participants were also asked about their fatigue symptoms over the past 6 months leading up to their index test date. In the follow-up questionnaires, participants reported on fatigue and PEM symptoms in relation to the last 14 days from when they responded to each questionnaire. This allowed for repeat collection of fatigue and PEM scores.

### Data Sources

EFTER-COVID survey data were collected using online Danish-language or English-language questionnaires produced in SurveyXact.^19^ All individual-level data from all data sources were linked using the CPR number in the Danish Civil Registration System, which encodes information on age and sex. We merged this information with participants’ responses to the questionnaires and information on comorbidities from the Danish National Patient Register (DNPR), which were used to calculate Charlson Comorbidity Index score, based on hospital contacts during the last 5 years before participants’ index test date. History of hospital-diagnosed psychiatric disorders between 2005 and up to the participants’ index test date was also captured from the DNPR; these included eating disorders, schizophrenia spectrum disorders, organic psychiatric disorders, anxiety disorders, stress-related disorders, depression, autism spectrum disorders, alcohol and other dependency-related disorders, attention-deficit/hyperactivity disorders, personality disorders, and bipolar disorders (eTable 1 and eTable 2 in Supplement 1). History of SARS-CoV-2 infection was ascertained from MiBa, acute SARS-CoV-2 hospitalization information was ascertained from the DNPR, and information on self-reported seropositivity and positive results from home rapid antigen testing was obtained from the EFTER-COVID survey. Health care workers were identified using the Authorization register^20^ and vaccination status from the Danish Vaccination Register.^21^ A complete description of variables and their data sources is available in eTable 1 in Supplement 1.

### Inclusion and Exclusion Criteria

Danish residents aged 15 years and older were eligible for participation in the EFTER-COVID survey. The study population included EFTER-COVID survey respondents who completed a baseline and at least 1 follow-up questionnaire 2 to 18 months after testing for SARS-CoV-2. Exclusions were applied on the basis of self-reported SARS-CoV-2 infection between the index test date and before completing the last follow-up questionnaire, as well as in cases where the first vaccine dose was received around the time of testing for SARS-CoV-2 (eFigure 2 in Supplement 1).

### Outcomes

Fatigue scores were determined using the FAS, where scores less than 22 indicate no fatigue, scores between 22 and 34 indicate mild-to-moderate fatigue, and scores of 35 or more indicate severe fatigue.^22,23^ These scores were examined as count and binary data. Binary categorizations captured (1) normal-range scores compared with mild-to-moderate or severe fatigue scores and (2) normal-range scores compared with severe fatigue scores. For PEM scores, a frequency and severity score of at least 2 and 2 on any items 1 to 5 was considered indicative of PEM.^24^

### Statistical Analysis

To compare repeated measurements of scores among individuals with SARS-CoV-2 infection and uninfected controls, we used Poisson mixed-effects models to estimate score ratios (SRs) with corresponding 95% CIs, which were calculated using the Wald method. Scores for fatigue between SARS-CoV-2 test-positive participants and test-negative participants were compared for (1) the combined 2 to 18 months of follow-up, (2) at different time points since testing (2, 4, 6, 9, 12, and 18 months since testing), and (3) 6 months preceding the index test, reported at baseline. These models took the following fixed effects into account: age, sex, BMI, Charlson Comorbidity Index score, health care occupation, SARS-CoV-2 variant, vaccination status, employment, and education level. The individual identifier was included as a random effect.

In models combining all measurements from the 2- to 18-month follow-up questionnaires, we examined factors predisposing postacute fatigue following SARS-CoV-2 infection by stratifying on hospitalization with acute SARS-CoV-2, age group, sex, BMI, Charlson Comorbidity Index score, health care occupation, dominant variant at test date, vaccination status, employment status, education attainment, preexisting asthma, chronic fatigue syndrome, fibromyalgia, and register-based and self-reported psychiatric disorders. These subgroup characteristics were explored according to associations identified in PCC literature^1,7^ and prior PCC risk groups identified previously.^25,26,27^ We also generated SRs across follow-up points between test-positive participants and test-negative participants, stratifying on (1) dominant SARS-CoV-2 variant at time of testing and (2) vaccination status at time of testing.

In addition, we used logistic mixed-effects models to estimate odds ratios (ORs) and 95% CIs 2 to 18 months after testing and at each follow-up point, comparing (1) mild-to-moderate and severe fatigue cases (FAS score ≥22, henceforth referred to as *substantial fatigue*) with noncases (FAS score <22) and (2) severe fatigue cases (FAS score ≥35) with noncases (FAS score <22). Models were also run comparing PEM cases (a frequency and severity score of at least 2 and 2 on any PEM items 1-5) and noncases (all other PEM scoring). Models were adjusted for the same fixed and random effects used in the aforementioned Poisson mixed-effects models.

Statistical significance was defined as a 95% CI that does not cross 1 (the null value). All statistical analyses were performed using R statistical software version 4.3.0 (R Project for Statistical Computing). The R package lme4^28^ was used for fitting mixed-effects models, and the forestploter package was used for generating forest plots.^29^

## Results

### Study Population

**Figure 1.**
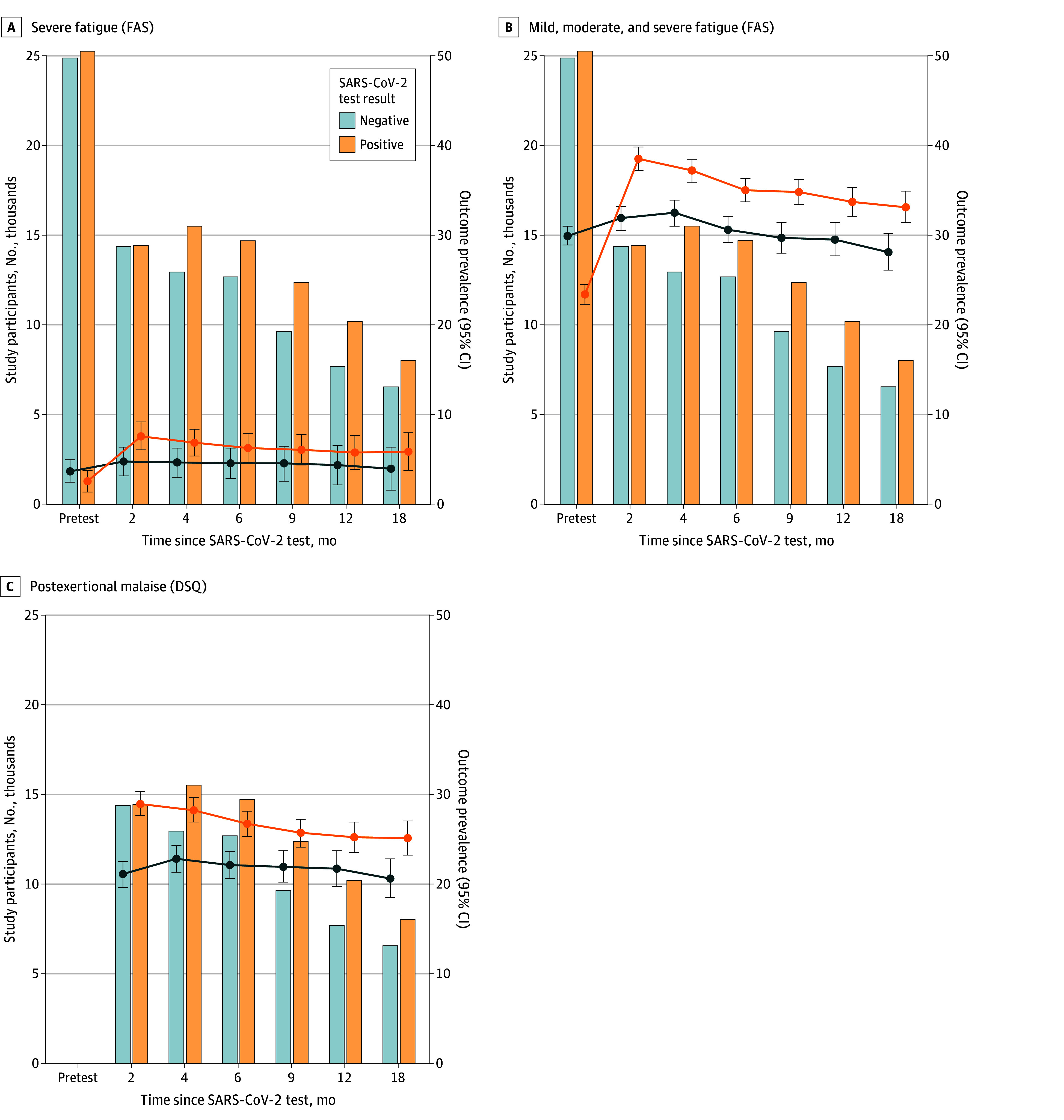
Prevalence of Fatigue and Postexertional Malaise Among Participants Graphs show number of study participants contributing with a questionnaire response at each time point divided on test result (bars, left y-axis) and prevalence of severe fatigue (defined as a score of 35-50 on the Fatigue Assessment Scale [FAS]), mild-to-moderate and severe fatigue (score of 22-50 on FAS), and postexertional malaise (both a frequency and severity score of at least 2 on any of the same items from 1-5) over time and by SARS-CoV-2 test result (lines and points, right y-axis; error bars denote 95% CIs). DSQ indicates DePaul Symptom Questionnaire.

Of the 75 695 individuals who had already completed the baseline questionnaire of the EFTER-COVID fatigue track and were invited to fill out at least 1 follow-up questionnaire, a total of 55 505 persons completed at least 1 follow-up questionnaire. After exclusions (5390 excluded participants) (eFigure 2 in Supplement 1), the study population consisted of 50 115 individuals (median [IQR] age at test date, 57 [46-67] years; 29 774 female [59.4%]), of whom 25 249 (50.4%) were test positive and 24 866 (49.6%) were test negative. The mean (SD) number of follow-up questionnaires (ie, not including baseline) completed was 2.6 (1.7) for test-negative participants and 3.0 (1.7) for test-positive participants (minimum, 1 questionnaire; maximum, 6 questionnaires) (Table). The number of responses to follow-up questionnaires generally decreased over time (Figure 1). Respondents were more frequently female than male, and the age group that accounted for the highest proportion of participants among both test-positive and test-negative participants was 50 to 69 years (Table). The index test date for the majority of test-positive and test-negative participants took place during the Omicron-dominant period. Most participants were vaccinated with at least 2 doses (21 164 test-negative participants [85.1%] and 22 120 test-positive participants [87.6%]) before their SARS-CoV-2 index test. Compared with nonparticipants, participants were more often older (median [IQR] age, 47 [32-58] years vs 57 [46-67] year) and had more comorbidities (Charlson Comorbidity Index score of ≥1, 2090 participants [10.4%] vs 7988 participants [14.4%]) (eTable 3 in Supplement 1).

**Table.** Overview of Study Population

Characteristic	Participants, No. (%) (N = 50 115)	
	Negative (n = 24 866)	Positive (n = 25 249)
No. of completed follow-up questionnaires		
Median (IQR)	2 (1-4)	3 (1-4)
Mean (SD)	2.6 (1.7)	3.0 (1.7)
Age group at test date, y		
15-29	1469 (5.9)	2455 (9.7)
30-49	4824 (19.4)	6988 (27.7)
50-69	12 929 (52.0)	11 273 (44.6)
≥70	5644 (22.7)	4533 (18.0)
Age at test date, median (IQR), y	60 (49-69)	55 (43-66)
Sex		
Female	14 734 (59.3)	15 040 (59.6)
Male	10 132 (40.7)	10 209 (40.4)
Body mass index^a^		
Without obesity	18 646 (75.0)	19 426 (76.9)
With obesity	4548 (18.3)	3972 (15.7)
Unknown	1672 (6.7)	1851 (7.3)
Charlson Comorbidity Index score		
0	20 772 (83.5)	22 056 (87.4)
1	2227 (9.0)	1874 (7.4)
≥2	1867 (7.5)	1319 (5.2)
Health care occupation		
No	22 636 (91.0)	23 507 (93.1)
Yes (frontline)	1603 (6.4)	1245 (4.9)
Yes (other)	627 (2.5)	497 (2.0)
Variant		
Omicron	10 192 (41.0)	12 946 (51.3)
Alpha	2830 (11.4)	1536 (6.1)
Delta	4296 (17.3)	3002 (11.9)
Intermediate transitional period	7548 (30.4)	7765 (30.8)
Vaccination status		
Unvaccinated	2534 (10.2)	2489 (9.9)
Vaccinated 1 dose	1168 (4.7)	640 (2.5)
Vaccinated 2 doses	9447 (38.0)	10 335 (40.9)
Vaccinated 3 doses	11 717 (47.1)	11 785 (46.7)
Employment		
Work and/or study (actively)	15 172 (61.0)	16 995 (67.3)
Work and/or study (not temporarily)	450 (1.8)	543 (2.2)
Work and/or study (not able)	317 (1.3)	277 (1.1)
Pensioner	7985 (32.1)	6304 (25.0)
Unknown	942 (3.8)	1130 (4.5)
Education		
Higher (long)	3963 (15.9)	4853 (19.2)
Higher (medium or short)	11 129 (44.8)	10 850 (43.0)
Secondary or vocational	6883 (27.7)	6436 (25.5)
Primary	2285 (9.2)	2194 (8.7)
Unknown	606 (2.4)	916 (3.6)

^a^ Body mass index is calculated as weight in kilograms divided by height in meters squared. Obesity was defined as body mass index of 30 or higher for individuals aged 18 years or older; for individuals aged 15 to 17 years, international cutoff points for obesity by sex and age were used.

For all participants, mean and median fatigue scores fell within the noncase range (FAS score <22) at all follow-up points (eTable 4 and eFigure 3 in Supplement 1). Reassuringly, mean (SD) pretest FAS scores in our test-negative population (19.4 [6.7]) were both below the case threshold (a score of ≥22) and aligned with prior mean score norms (19.3).^22^ For test-positive participants, the prevalence of severe fatigue (1102 participants [7.6%]), substantial fatigue (5554 participants [38.5%]), and PEM (4174 participants [28.9%]) was highest at the 2-month follow-up point and generally decreased over time. At the 18-month follow-up, the prevalence of severe fatigue was 5.9% (474 participants), that of substantial fatigue was 33.1% (2659 participants), and that of PEM was 25.1% (2014 participants) (Figure 1 and eTable 5 in Supplement 1).

### Risk of Fatigue

**Figure 2.**
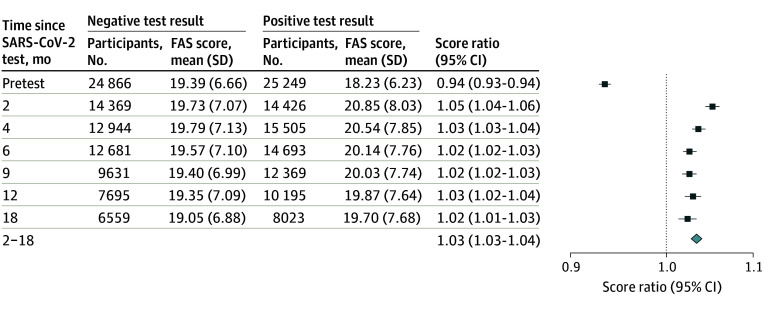
Score Ratios and 95% CIs for Fatigue Assessment Scale (FAS) Scores Between Test-Positive and Test-Negative Individuals Estimates are presented for pretest scores (ie, 6 months before the index test), each follow-up point, and the combined 2 to 18 months after testing. Poisson mixed-effects models took the following fixed effects into account: age, sex, body mass index (calculated as weight in kilograms divided by height in meters squared; defined as with obesity [≥30], without obesity [<30], and unknown or missing), Charlson Comorbidity Index score (0, 1, or ≥2), health care occupation, SARS-CoV-2 variant (Alpha, Delta, Omicron, or Transition), vaccination status (unvaccinated and 1, 2, or 3 doses), employment, and education level. The individual identifier was included as a random effect. Diamond denotes score ratio and 95% CI over the combined 2- to 18-month period.

In the period 2 to 18 months after testing, test-positive participants had a significant 3% increase in scores for fatigue compared with test-negative participants (SR, 1.03; 95% CI, 1.03-1.04). Significant increases in scores associated with testing positive were also observed at the follow-ups at 2, 4, 6, 9, 12, and 18 months. However, these increases, although statistically significant, still showed mean scores that were within the noncase range (Figure 2). When dichotomizing outcomes, test-positive participants had almost twice the odds (OR, 1.93; 95% CI, 1.64-2.26) of severe fatigue (scores ≥35; reference, score <22) compared with test-negative participants 2 to 18 months after testing (eFigure 4 in Supplement 1). Higher odds were also observed for test-positive participants for substantial fatigue (scores ≥22; reference, score <22) 2 to 18 months after testing (OR, 1.63; 95% CI, 1.52-1.75) (eFigure 5 in Supplement 1).

**Figure 3.**
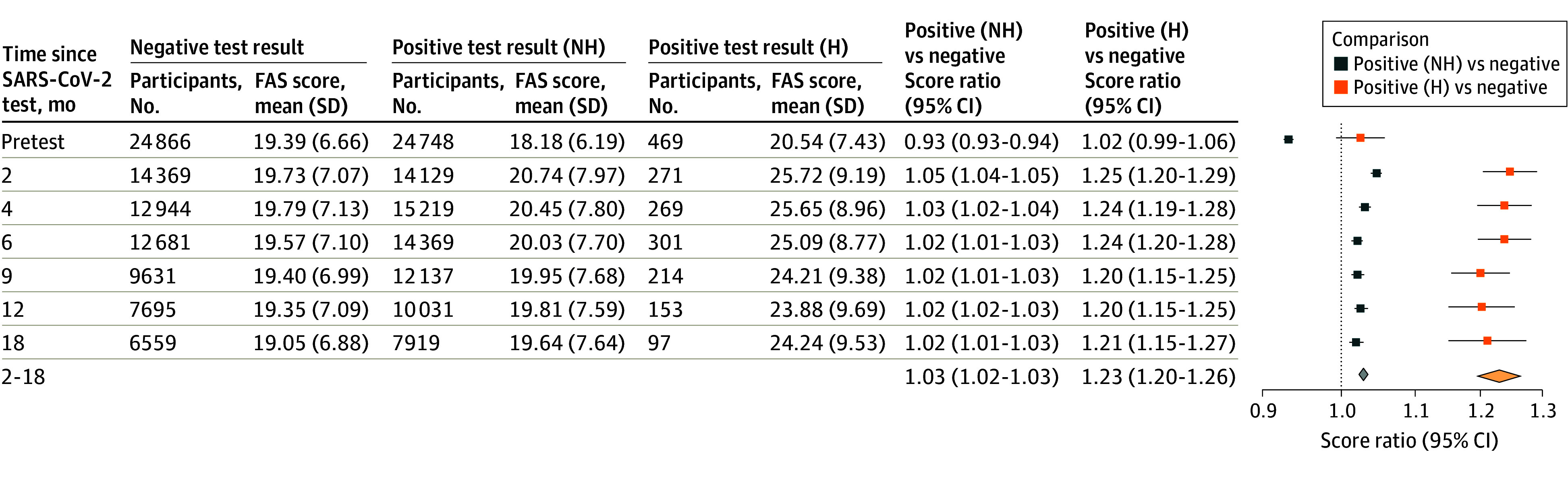
Score Ratios and 95% CIs for Fatigue Assessment Scale (FAS) Scores Between Nonhospitalized (NH) and Acutely Hospitalized (H) Test-Positive and Test-Negative Individuals Estimates are presented for pretest scores (ie, 6 months before the index test), each follow-up point, and the combined 2-18 months after testing. Poisson mixed-effects models took the following fixed effects into account: age, sex, body mass index (calculated as weight in kilograms divided by height in meters squared; defined as with obesity [≥30], without obesity [<30], and unknown or missing), Charlson Comorbidity Index score (0, 1, or ≥2), health care occupation, SARS-CoV-2 variant (Alpha, Delta, Omicron, or Transition), vaccination status (unvaccinated and 1, 2, or 3 doses), employment, and education level. The individual identifier was included as a random effect. Thirty-two individuals who tested positive more than 2 days after hospitalization based on other reasons were excluded. Diamond denotes score ratio and 95% CI over the combined 2- to 18-month period.

**Figure 4.**
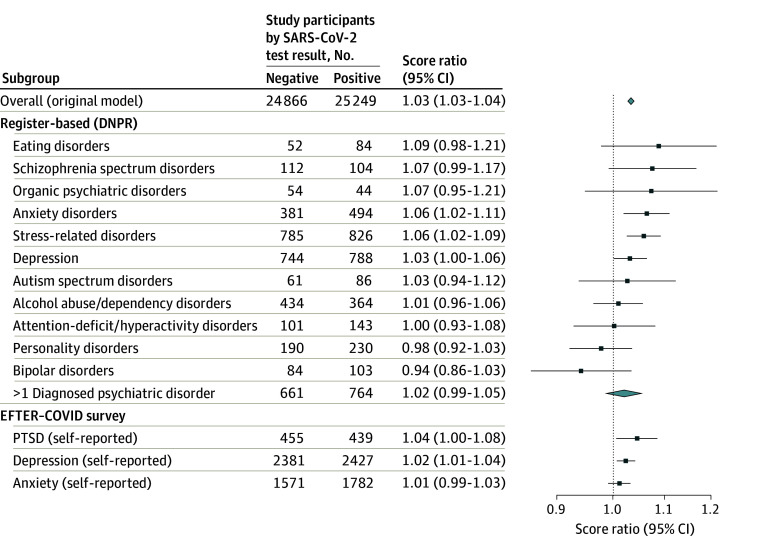
Score Ratios and 95% CIs for Fatigue Assessment Scale (FAS) Scores 2 to 18 Months After Testing Between Test-Positive and Test-Negative Patients, Stratified by Psychiatric Conditions Poisson mixed-effects models took the following fixed effects into account: age, sex, body mass index (calculated as weight in kilograms divided by height in meters squared; defined as with obesity [≥30], without obesity [<30], and unknown or missing), Charlson Comorbidity Index score (0, 1, or ≥2), health care occupation, SARS-CoV-2 variant (Alpha, Delta, Omicron, or Transition), vaccination status (unvaccinated and 1, 2, and 3 doses), employment, and education level. The individual identifier was included as a random effect. DNPR indicates Danish National Patient Register; EFTER COVID, AFTER COVID; and PTSD, posttraumatic stress disorder. Diamond denotes score ratio and 95% CI over the combined 2- to 18-month period in the overall study population.

Persons hospitalized with acute SARS-CoV-2 infection had a notable 23% increase in scores (SR, 1.23; 95% CI, 1.20-1.26) compared with test-negative participants 2 to 18 months after testing, and hospitalized individuals’ mean scores fell within the mild-to-moderate fatigue range (Figure 3). Stratified analyses showed that SARS-CoV-2 infection was associated with increased FAS scores 2 to 18 months after testing among those with high BMI (SR, 1.06; 95% CI, 1.05-1.08) and among those who tested during the Alpha-dominant variant (SR, 1.09; 95% CI, 1.07-1.11) (eFigure 6 in Supplement 1). Notably, when comparing test-positive with test-negative participants, persons infected during the Alpha wave reported higher fatigue scores across follow-up points compared with corresponding SRs during the Omicron wave (eFigure 7 in Supplement 1). Among individuals unvaccinated or vaccinated with only 1 dose, SRs were slightly higher in several follow-up points compared with those vaccinated with 2 or 3 doses (eFigure 8 in Supplement 1). Of the 11 register-based and 3 self-reported preexisting psychiatric conditions, none significantly modified the risk of self-reported postacute fatigue 2 to 18 months after testing. Having multiple preexisting psychiatric conditions also did not significantly impact the risk of self-reported postacute fatigue during this period (Figure 4).

### Risk of PEM

Compared with test-negative participants, test-positive participants had 2 times higher odds of experiencing PEM (OR 2.04; 95% CI, 1.81-2.30) in the 2- to 18-month period after testing. Odds of PEM were highest for test-positive participants at 2 months after testing (OR 2.92; 95% CI, 2.52-3.39), compared with test-negative participants. Odds of PEM were significantly higher for test-positive participants than test-negative participants across all follow-up points (eFigure 9 in Supplement 1).

## Discussion

This cohort study found that SARS-CoV-2 infections were associated with elevated risk of self-reported chronic fatigue and PEM symptoms in the period 2 to 18 months after infection, but the magnitude of change in fatigue scores was low. Of the possible predisposing factors explored, acute SARS-CoV-2 hospitalization had the greatest impact on fatigue scores. This finding corroborates much of the literature underscoring that severe SARS-CoV-2 infection is associated with worse PCC outcomes.^1,7^ Contrary to reports examining dichotomous PCC outcomes,^7,8,9^ we found that preexisting psychiatric conditions did not significantly alter the risk of PCC fatigue. Our findings should be considered in the context of a mostly vaccinated (2-3 doses) study population, as vaccination has been shown to protect against PCC^7^ and specifically PCC fatigue.^30^

A study^31^ conducted in Washington State also examined the incidence of and factors associated with post–COVID-19 fatigue using a study population of 4589 confirmed pre-Delta and pre-Omicron SARS-CoV-2 cases and 9022 propensity score–matched uninfected controls. Among nonhospitalized patients with SARS-CoV-2, the incidence of PCC fatigue was 10% per year, where patients with SARS-CoV-2 had 4.32 times the risk of chronic fatigue diagnosis compared with uninfected controls. The authors also observed that patients with a history of mood disorders were at an increased risk of PCC fatigue, compared with persons without mood disorders. In their study, infections largely occurred before vaccine rollout, and information on vaccination was not available.^31^ Another US study^32^ capturing SARS-CoV-2 tests from December 2020 to 2023 noted that extreme fatigue first emerged 6 months after testing. Our study findings reiterate an increased risk of fatigue after infection but capture a study population from a later pandemic stage, when most patients with SARS-CoV-2 were vaccinated before infection. The observed incidence of fatigue was less pronounced in our study owing to vaccine protection preceding infection. We observed a greater risk of PCC fatigue symptoms among individuals who tested positive during the Alpha wave, when most were not yet vaccinated. Persons who had received no or only 1 vaccine dose before testing had higher SRs across several follow-up points, compared with persons who had received 2 or 3 doses. Although individuals with a history of hospital contact for anxiety and stress-related disorders also experienced an increased risk of fatigue after infection, overlapping 95% CIs suggested that these conditions did not further increase risk of PCC fatigue, and results for self-reported psychiatric conditions were not significant. This corroborates findings from a Bavarian study,^13^ which also used a noninfected control group to examine the associations between preexisting psychiatric conditions and PCC. The authors found that persons with preexisting mental disorders had the same or even greater risk of fatigue or malaise diagnosis in the noninfected control cohort (hazard ratio [HR], 1.71; 95% CI, 1.52-1.93) and other respiratory infection cohort (HR, 1.43; 95% CI, 1.20-1.72), than in the COVID-19 cohort (HR, 1.43; 95% CI, 1.35-1.51).^13^ Furthermore, a multinational Nordic study^14^ found that SARS-CoV-2 hospitalization was a key risk factor for PCC fatigue, as did our findings. A systematic review^10^ of post–COVID-19 fatigue outcomes pointed to fatigue rates decreasing by 6% per month after infection but highlighted dependence on when fatigue was measured. In our study, for all measures of fatigue and PEM, risk was highest 2 months after testing and, although present, had decreased by the 18-month follow-up point.

To our knowledge, this study is among the largest survey investigations of PCC fatigue using validated measures capturing both physical and mental symptoms of chronic fatigue^22,33^ and PEM, a key symptom of myalgic encephalomyelitis and chronic fatigue syndrome.^24^ Furthermore, the investigation of fatigue outcomes as both count and binary data delivers a nuanced understanding of changes in risk. For instance, significantly higher odds of fatigue outcomes were observed for infected compared with noninfected persons upon using normed, dichotomized cutoffs for severe and mild-to-moderate fatigue cases; however, a more granular examination treating scores as count data demonstrated a subtle (3%) increase in fatigue scores and normal-range mean scores associated with infection. This suggests a low magnitude of change posed by infection in a mostly vaccinated cohort. When dichotomizing a scale, moderate increases in the odds will arise from subtle increases in scores. Results from dichotomized analyses should, therefore, be interpreted carefully. Finally, the FAS scores observed in this study are derived from subjective, anonymous reporting of physical and mental fatigue symptoms, where we do not see significant differences for long-term, severe fatigue (scores 35-50) between test-positive participants compared with test-negative participants. Clinical, objective, in-person assessments of fatigue severity in patients with suspected PCC could demonstrate different results.

### Limitations

The study’s limitations must be carefully considered. First, the observed estimates could be influenced by uncontrollable factors such as unknown or undetected SARS-CoV-2 infections in the test-negative control group or unknown reinfections in the test-positive cohort. In addition, we did not gather PEM scores corresponding to the 6 months before testing, so we could not examine pre–SARS-CoV-2 exposure scores. The prevalence of PEM indication in the study population appears quite high across follow-up points but only captures the first stage of identifying PEM, as second-stage questions^24^ were not asked in the survey. The absence of PEM scoring at baseline and these second-stage questions limits the specificity of the present findings and overall understanding of the incidence of PCC PEM. Finally, we cannot rule out bias introduced by some participants receiving their baseline questionnaire at differing months after testing, which resulted from administrative error.

## Conclusions

In conclusion, SARS-CoV-2 infection was associated with a subtle increase in the risk of postacute fatigue and PE 2 to 18 months after mild infection. These findings reflect outcomes in a mostly vaccinated study population. Although preexisting psychiatric conditions were not associated with further increases in the risk of postacute fatigue, persons hospitalized for acute SARS-CoV-2 had a greater risk.

## Notes

Supplement 1. **eTable 1.** Overview of study variables **eFigure 1.** Overview of study design **eFigure 2.** Flowchart for selection of study population **eTable 2.** *ICD-10 *codes for register-based psychiatric disorders **eTable 3.** Table including study characteristics (test result, age group at test date, age numeric at test date, sex, BMI, CCI, healthcare occupation, variant, vaccination status, employment, and education) stratified by follow-up study participation **eTable 4.** Mean and median Fatigue Assessment Scale (FAS) scores leading up to the test and at each follow-up point, by SARS-CoV-2 test result **eTable 5.** Prevalence of severe fatigue, mild/moderate fatigue, and post-exertional malaise for SARS-CoV-2 test-positives and -negatives over time **eFigure 3.** Distribution of Fatigue Assessment Scale (FAS) scores leading up to the test and at each follow-up point, by SARS-CoV-2 test result **eFigure 4.** Odds ratios (OR) and 95% confidence intervals (CI) for severe fatigue scores (scores ≥35, ref: <22) between test-positive- and negative individuals **eFigure 5.** Odds ratios (OR) and 95% confidence intervals (CI) for substantial fatigue scores (≥22, ref: <22) between test-positive- and negative individuals **eFigure 6.** Score ratios (SR) and 95% confidence intervals (CI) for FAS scores 2-18 months after testing between test-positives and -negatives, stratified by possible risk factors **eFigure 7.** Score ratios (SRs) and 95% confidence intervals (CI) for FAS scores across follow-up points after testing between test-positives and -negatives, stratified by dominant SARS-CoV-2 variant at time of index testing **eFigure 8.** Score ratios (SRs) and 95% confidence intervals (CI) for FAS scores 2-18 months after testing between test-positives and -negatives, stratified by vaccination status at time of SARS-CoV-2 index test **eFigure 9.** Odds ratios (OR) and 95% confidence intervals (CI) for post-exertional malaise (PEM) (frequency and severity score of at least 2 and 2 on any items, REF: all other scoring) between test-positive- and negative individuals

Supplement 2.
Data Sharing Statement
