# Supplementary material for: Post–COVID-19 Condition Fatigue Outcomes Among Danish Residents
Source: JAMA Netw Open. 2024 Oct 7;7(10):e2434863. doi: 10.1001/jamanetworkopen.2024.34863 (PMC11581651; doi:10.1001/jamanetworkopen.2024.34863)
Supplement: Supplement 1. — eTable 1. Overview of study variables eFigure 1. Overview of study design eFigure 2. Flowchart for selection of study population eTable 2. ICD-10 codes for register-based psychiatric disorders eTable 3. Table including study characteristics (test result, age group at test date, age numeric at test date, sex, BMI, CCI, healthcare occupation, variant, vaccination status, employment, and education) stratified by follow-up study participation eTable 4. Mean and median Fatigue Assessment Scale (FAS) scores leading up to the test and at each follow-up point, by SARS-CoV-2 test result eTable 5. Prevalence of severe fatigue, mild/moderate fatigue, and post-exertional malaise for SARS-CoV-2 test-positives and -negatives over time eFigure 3. Distribution of Fatigue Assessment Scale (FAS) scores leading up to the test and at each follow-up point, by SARS-CoV-2 test result eFigure 4. Odds ratios (OR) and 95% confidence intervals (CI) for severe fatigue scores (scores ≥35, ref: <22) between test-positive- and negative individuals eFigure 5. Odds ratios (OR) and 95% confidence intervals (CI) for substantial fatigue scores (≥22, ref: <22) between test-positive- and negative individuals eFigure 6. Score ratios (SR) and 95% confidence intervals (CI) for FAS scores 2-18 months after testing between test-positives and -negatives, stratified by possible risk factors eFigure 7. Score ratios (SRs) and 95% confidence intervals (CI) for FAS scores across follow-up points after testing between test-positives and -negatives, stratified by dominant SARS-CoV-2 variant at time of index testing eFigure 8. Score ratios (SRs) and 95% confidence intervals (CI) for FAS scores 2-18 months after testing between test-positives and -negatives, stratified by vaccination status at time of SARS-CoV-2 index test eFigure 9. Odds ratios (OR) and 95% confidence intervals (CI) for post-exertional malaise (PEM) (frequency and severity score of at least 2 and 2 on any items, REF: all other scoring) between test-p [file jamanetwopen-e2434863-s001.pdf]

Supplemental Online Content

O'Regan E, Spiliopoulos L, Svalgaard IB, et al. Post–COVID-19 condition fatigue outcomes among Danish residents. *JAMA Netw Open*. 2024;7(9):e2434863. doi:10.1001/jamanetworkopen.2024.34863

eTable 1. Overview of study variables

eFigure 1. Overview of study design

eFigure 2. Flowchart for selection of study population

eTable 2. *ICD-10* codes for register-based psychiatric disorders

eTable 3. Table including study characteristics (test result, age group at test date, age numeric at test date, sex, BMI, CCI, healthcare occupation, variant, vaccination status, employment, and education) stratified by follow-up study participation

eTable 4. Mean and median Fatigue Assessment Scale (FAS) scores leading up to the test and at each follow-up point, by SARS-CoV-2 test result

eTable 5. Prevalence of severe fatigue, mild/moderate fatigue, and post-exertional malaise for SARS-CoV-2 test-positives and -negatives over time

eFigure 3. Distribution of Fatigue Assessment Scale (FAS) scores leading up to the test and at each follow-up point, by SARS-CoV-2 test result

eFigure 4. Odds ratios (OR) and 95% confidence intervals (CI) for severe fatigue scores (scores ≥35, ref: <22) between test-positive- and negative individuals

eFigure 5. Odds ratios (OR) and 95% confidence intervals (CI) for substantial fatigue scores (≥22, ref: <22) between test-positive- and negative individuals

eFigure 6. Score ratios (SR) and 95% confidence intervals (CI) for FAS scores 2-18 months after testing between test-positives and -negatives, stratified by possible risk factors

eFigure 7. Score ratios (SRs) and 95% confidence intervals (CI) for FAS scores across follow-up points after testing between test-positives and -negatives, stratified by dominant SARS-CoV-2 variant at time of index testing

© 2024 O'Regan E et al. *JAMA Network Open.*

eFigure 8. Score ratios (SRs) and 95% confidence intervals (CI) for FAS scores 2-18 months after testing between test-positives and -negatives, stratified by vaccination status at time of SARS-CoV-2 index test

eFigure 9. Odds ratios (OR) and 95% confidence intervals (CI) for post-exertional malaise (PEM) (frequency and severity score of at least 2 and 2 on any items, REF: all other scoring) between test-positive- and negative individuals

This supplemental material has been provided by the authors to give readers additional information about their work.

© 2024 O'Regan E et al. *JAMA Network Open.*

eTable 1: Overview of study variables.

Name	Description	Data type and variable level description	Categorization	Data source
Fatigue	Self-reported. Based on questions from ©FAS	Ordinal (scale 10-50)<br><br>10-21: normal<br>22-34: mild/moderate fatigue<br>35-50: severe fatigue	1) Count*<br><br>2) Binary (10-21 = No, 22-50 = Yes**)<br><br>3) Binary (<22 = No, 35-50 = Yes**)	EFTER-COVID
Post-exertional malaise	Self-reported. Based on questions from DSQ.	Binary<br>Both a frequency and severity score of 2 on any of the same items from 1-5: indicative of PEM<br>Scoring otherwise: Normal	2) Binary (scoring otherwise = No, pairwise scores of ≥2 on any of the items from 1-5 = Yes**)	EFTER-COVID
SARS-CoV-2 test result	Index test initiating invitation to EFTER-COVID survey. Registered.	Binary	Positive, negative	MiBa
Age group	Age calculated at the index test date	Categorical	15-29, 30-49, 50-69, 70+	CPR registry
Sex	Sex assigned at birth	Binary	Female, Male	CPR registry
BMI	Self-reported. Based on questions from the baseline questionnaire (non-obligatory information)	Categorical<br><br>Obese: BMI≥30 for individuals aged 18 years or above; for 15-17-year olds international cut-off points for obesity by sex and age were used<br>Non-obese	Obese, Non-obese, Unknown	EFTER-COVID

		Unknown: missing height or weight		
Charlson Comorbidity Index	Based on hospital contacts 5 years prior and up to the index test date	Categorical	0, 1, ≥2	DNPR
Healthcare occupation	Based on information at the index test date	Categorical<br><br>Frontline: Doctor, Nurse, Health/social assistant<br>Other: Ambulance attendant, Bandage specialist, Treating pharmacist, Medical laboratory technologist, Occupational therapist, Podiatrist, Physiotherapist, Midwife, Chiropractor, Clinical dietitian, Clinical dental technician, Optician, Optometrist, Osteopath, Radiographer, Dentist, Dental therapist	No, Yes (frontline), Yes (other)	Authorisation registry
Variant	Period of variant predominance	Categorical<br><br>Alpha: 15/03/2021 - 30/06/2021<br>Delta: 15/07/2021 - 15/11/2021<br>Omicron: 01/01/2022 - 21/02/2023 (last study test date)<br>Transition: intermediary transitional periods	Alpha, Delta, Omicron, Transition	Michlmayr et. al
Vaccination status	Based on information at the index test date	Categorical	Unvaccinated, Vaccinated (1 dose), Vaccinated (2 doses), Vaccinated (3 doses)	Danish Vaccination Register

© 2024 O'Regan E et al. *JAMA Network Open.*

Employment	Self-reported at the index test date	Categorical<br><br>Work/study (actively): Employed full/part time, Self-employed, Student<br>Work/study (not active): Jobseeking/Unemployed, Stay-at-home parent or on parental leave<br>Work/study (not able): Benefits recipient, Long-term sick leave<br>Pensioner: Pensioner<br>Unknown: Other, Missing	Work/study (actively), Work/study (not temporarily), Work/study (not able), Pensioner, Unknown	EFTER-COVID
Education	Self-reported at the index test date	Categorical<br><br>Higher (long): Longer term higher education (more than 5 years), e.g. Masters or PhD,<br>Higher (medium/short): Medium term higher education (2-4 years), e.g. nursing, primary school teaching, BSc, Shorter term higher education (1-2 years), e.g. vocational academy<br>Secondary/vocational: General secondary education or vocational secondary education, Vocational training<br>Primary: Primary/Elementary school (9th-10th grade)<br>Unknown: Do not know or None of the above or Do	Higher (long), Higher (medium/short), Secondary/vocational, Primary, Unknown	EFTER-COVID

© 2024 O'Regan E et al. *JAMA Network Open.*

		not wish to answer, Missing		
History of psychiatric conditions	Based on registered ICD-10 code diagnoses from hospitalizations between 2005 and participants' index test date	Categorical	Eating disorders, schizophrenia spectrum disorders, organic psychiatric disorders, anxiety disorders, stress-related disorders, depression, autism spectrum disorders, substance use disorders, attention-deficit/hyperactivity disorders, personality disorders, and bipolar disorders, >1 psychiatric condition, none	DNPR
Self-reported conditions	Self-reported at the index date	Categorical	post-traumatic stress disorder, depression, anxiety, asthma, chronic fatigue syndrome, fibromyalgia, none	EFTER-COVID
Acute SARS-CoV-2 hospitalization	Registered	Categorical	Non-hospitalized, hospitalized (positive test within 14 days of admission or positive test up to 48 hours after admission), unclear hospitalization (admitted and tested 48 hours or later after hospitalization, indicative of hospital-acquired SARS-CoV-2)	DNPR

©FAS: Fatigue Assessment Scale
DSQ: Depaul Symptom Questionnaire
PEM : Post-exertional malaise
*Used in Poisson mixed effects models
**Used in logistic mixed effects models

© 2024 O'Regan E et al. *JAMA Network Open.*

eFigure 1: Overview of study design. A. Design of EFTER-COVID survey. B. Variables obtained from survey and register data.

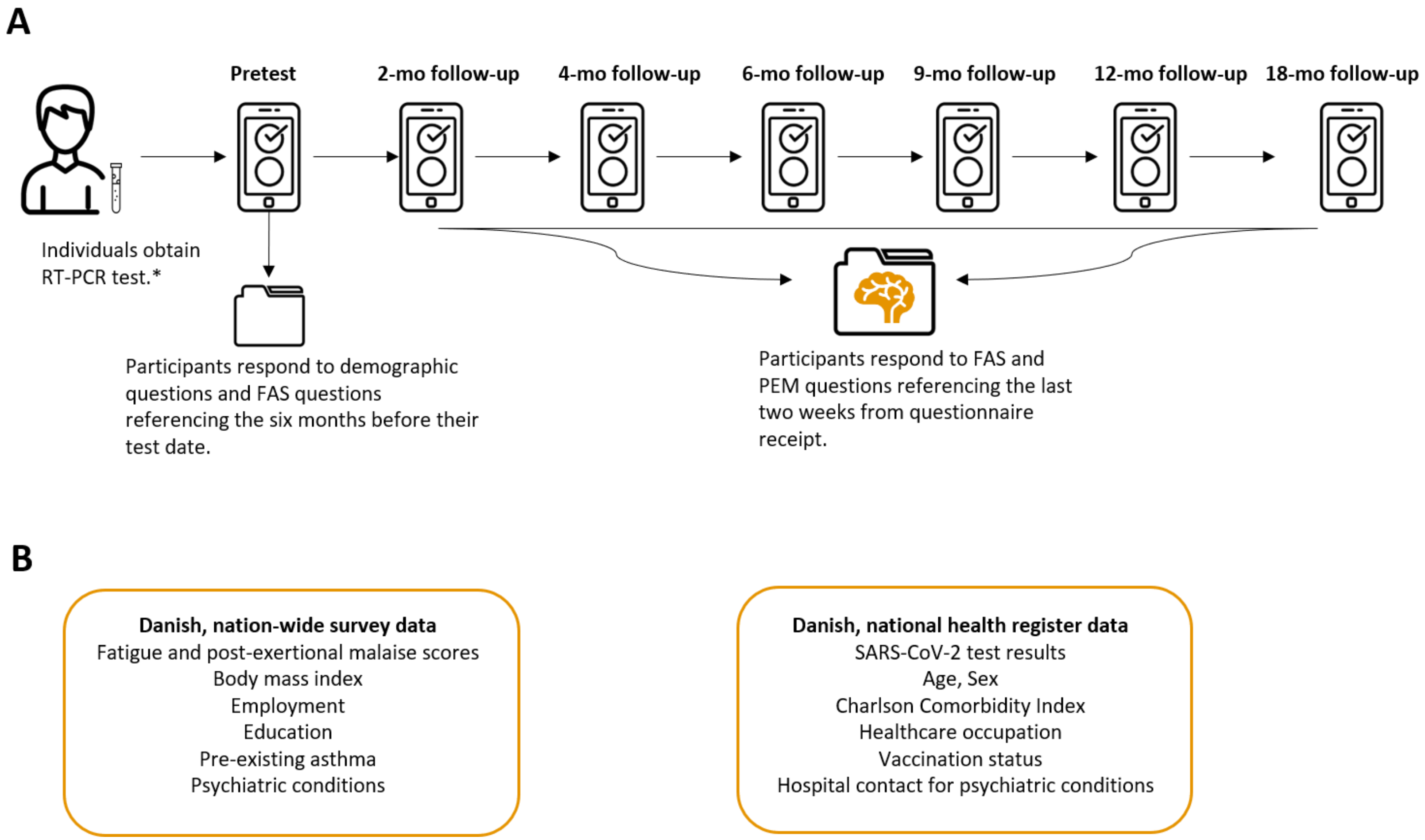

FAS: ©Fatigue Assessment Scale, PEM : Post-exertional malaise

© 2024 O'Regan E et al. *JAMA Network Open.*

eFigure 2: Flowchart for selection of study population.

n = 75,695 EFTER-COVID participants of the fatigue study track who had already completed the baseline questionnaire, received an invitation to complete at least one follow-up questionnaire 2-18 months after testing for SARS-CoV-2 by October 4th, 2023

Negative: n = 40,544
Positive: n = 35,151

↓

n = 55,505 EFTER-COVID participants completed at least one follow-up questionnaire 2-18 months after testing for SARS-CoV-2

Negative: n = 29,906 (follow-up participation rate: 73.8%)
Positive: n = 25,599 (follow-up participation rate: 72.8%)

→ Excluded participants (n = 5,390)

1) n = 4,424 test-negative EFTER-COVID participants who reported testing positive between the test date and the time of completing the last follow-up questionnaire.

2) n = 966 individuals who received the first vaccination dose within 14 days prior to testing for SARS-CoV-2, i.e. without full effect of first vaccination dose at the test date.

↓

n = 50,115 EFTER-COVID participants who completed the baseline questionnaire and at least one follow-up questionnaire 2-18 months after testing for SARS-CoV-2, comprised the final study population

Negative: n = 24,866
Positive: n = 25,249

© 2024 O’Regan E et al. *JAMA Network Open.*

eTable 2: *ICD-10* codes for register-based psychiatric disorders.

Disorder	ICD-10 code	Text
Alcohol abuse, dependency, and related disorders	F10	Mental and behavioral disorders (MBD) due to use of alcohol
	F11	MBD due to use of opioids
	F12	MBD due to use of cannabinoids
	F13	MBD due to use of sedatives or hypnotics
	F14	MBD due to use of cocaine
	F15	MBD due to use of other stimulants, including caffeine
	F16	MBD due to use of hallucinogens
	F18	MBD due to use of volatile solvents
	F19	MBD due to multiple drug use and use of other psychoactive substances
	E24.4	Alcohol-induced pseudo-Cushing syndrome
	G31.2	Degeneration of nervous system due to alcohol
	G62.1	Alcoholic polyneuropathy
	G72.1	Alcoholic myopathy
	I42.6	Alcoholic cardiomyopathy
	K29.2	Alcoholic gastritis
	K70	Alcoholic liver disease
	K85.2	Alcohol-induced acute pancreatitis
	K86.0	Alcohol-induced chronic pancreatitis
	Q86.0	Fetal alcohol syndrome (dysmorphic)
Organic psychiatric disorders	F06	Other mental disorders due to brain damage and dysfunction and to physical disease
	F07	Personality and behavioral disorders due to brain disease, damage and dysfunction
Schizophrenia spectrum disorders	F20	Schizophrenia
	F21	Schizotypal disorder
	F22	Persistent delusional disorders
	F23	Acute and transient psychotic disorders
	F24	Induced delusional disorder
	F25	Schizoaffective disorders
	F28	Other nonorganic psychotic disorders
	F29	Unspecified nonorganic psychosis
Bipolar disorders	F30	Manic episode
	F31	Bipolar affective disorder
Depression	F32	Depressive episode
	F33	Recurrent depressive disorder
Anxiety disorders	F40	Phobic anxiety disorders
	F41	Other anxiety disorders
	F42	Obsessive-compulsive disorder
Stress-related disorders	F43	Reaction to severe stress, and adjustment disorders
Eating disorders	F50.0	Anorexia nervosa
	F50.1	Atypical anorexia nervosa
	F50.2	Bulimia nervosa
	F50.3	Atypical bulimia nervosa
Personality disorders	F60	Specific personality disorders
Autism spectrum disorder	F84.0	Childhood autism
	F84.1	Atypical autism
	F84.5	Asperger syndrome

© 2024 O'Regan E et al. *JAMA Network Open.*

	F84.8<br>F84.9	Other pervasive developmental disorders<br>Pervasive developmental disorder, unspecified
Attention-deficit/ hyper-activity disorder	F90<br>F98.8	Hyperkinetic disorders<br>Other specified behavioral and emotional disorders with onset usually occurring in childhood and adolescence

eTable 3: Table including study characteristics (test result, age group at test date, age numeric at test date, sex, BMI, CCI, healthcare occupation, variant, vaccination status, employment, and education) stratified by follow-up study participation.

	Invited to follow-up (n=75695)	
	**Participants n=55505**	**Non-participants n=20190**
**Test result (n, %)**		
Negative	29906 (53.9)	10638 (52.7)
Positive	25599 (46.1)	9552 (47.3)
**Age group at test date (n, %)**		
15-29	4336 (7.8)	4203 (20.8)
30-49	12981 (23.4)	7280 (36.1)
50-69	27234 (49.1)	7077 (35.1)
70+	10954 (19.7)	1630 (8.1)
**Age at test date**		
Median (IQR)	57 (46, 67)	47 (32, 58)
**Sex (n,%)**		
Female	33195 (59.8)	11975 (59.3)
Male	22310 (40.2)	8215 (40.7)
**BMI (n, %)**		
Non-obese	42270 (76.2)	15560 (77.1)
Obese	9420 (17.0)	3096 (15.3)
Unknown	3815 (6.9)	1534 (7.6)
**Charlson comorbidity index (n, %)**		
0	47517 (85.6)	18100 (89.6)
1	4506 (8.1)	1209 (6.0)
≥2	3482 (6.3)	881 (4.4)
**Healthcare occupation (n, %)**		
No	50967 (91.8)	18605 (92.1)
Yes (frontline)	3272 (5.9)	1162 (5.8)
Yes (other)	1266 (2.3)	423 (2.1)
**Variant (n, %)**		
Omicron	25472 (45.9)	9919 (49.1)
Alpha	5225 (9.4)	1765 (8.7)
Delta	7950 (14.3)	2614 (12.9)
Intermediate transitional period	16858 (30.4)	5892 (29.2)
**Vaccination status (n, %)**		
Unvaccinated	6276 (11.3)	2796 (13.8)
Vaccinated (1 dose)	1890 (3.4)	724 (3.6)
Vaccinated (2 doses)	21317 (38.4)	8938 (44.3)
Vaccinated (3 doses)	26022 (46.9)	7732 (38.3)
**Employment (n, %)**		
Work/study (actively)	35879 (64.6)	15876 (78.6)
Work/study (not temporarily)	1120 (2.0)	587 (2.9)
Work/study (not able)	647 (1.2)	351 (1.7)
Pensioner	15601 (28.1)	2601 (12.9)
Unknown	2258 (4.1)	775 (3.8)

© 2024 O'Regan E et al. *JAMA Network Open.*

**Education (n, %)**		
Higher (long)	10028 (18.1)	3636 (18.0)
Higher (medium or short)	24486 (44.1)	7719 (38.2)
Secondary or vocational	14560 (26.2)	5773 (28.6)
Primary	4821 (8.7)	2338 (11.6)
Unknown	1610 (2.9)	724 (3.6)

Body Mass Index (BMI): Obesity was defined as BMI≥30 for individuals aged 18 years or above; for 15-17-year olds international cut-off points for obesity by sex and age were used.

© 2024 O'Regan E et al. *JAMA Network Open.*

eTable 4: Mean and median Fatigue Assessment Scale (FAS) scores leading up to the test and at each follow-up point, by SARS-CoV-2 test result.

Time since SARS-CoV-2 test (months)	Test result	FAS total score (min:10, max:50)	
		Median (IQR)	Mean (SD)
Pre-test *	Negative	18.00 (15.00, 23.00)	19.39 (6.66)
Pre-test *	Positive	17.00 (14.00, 21.00)	18.23 (6.23)
2	Negative	18.00 (15.00, 23.00)	19.73 (7.07)
2	Positive	19.00 (15.00, 25.00)	20.85 (8.03)
4	Negative	19.00 (15.00, 23.00)	19.79 (7.13)
4	Positive	19.00 (15.00, 25.00)	20.54 (7.85)
6	Negative	18.00 (14.00, 23.00)	19.57 (7.10)
6	Positive	19.00 (14.00, 24.00)	20.14 (7.76)
9	Negative	18.00 (14.00, 23.00)	19.40 (6.99)
9	Positive	19.00 (14.00, 24.00)	20.03 (7.74)
12	Negative	18.00 (14.00, 23.00)	19.35 (7.09)
12	Positive	18.00 (14.00, 24.00)	19.87 (7.64)
18	Negative	18.00 (14.00, 22.00)	19.05 (6.88)
18	Positive	18.00 (14.00, 24.00)	19.70 (7.68)

** Pre-test refers to the six-month period prior and up to the index test date*
IQR: Interquartile range
SD: Standard deviation

© 2024 O'Regan E et al. *JAMA Network Open.*

eTable 5: Prevalence of severe fatigue, mild/moderate fatigue, and post-exertional malaise for SARS-CoV-2 test-positives and -negatives over time.

Outcome	Months since SARS-CoV-2 test	Negative n (%)	Positive n (%)	Negative n (participants)	Positive n (participants)
Severe Fatigue (FAS ≥ 35)	Pre-test *	929 (3.7)	663 (2.6)	24866	25249
	2	691 (4.8)	1102 (7.6)	14369	14426
	4	602 (4.7)	1070 (6.9)	12944	15505
	6	584 (4.6)	925 (6.3)	12681	14693
	9	438 (4.6)	754 (6.1)	9631	12369
	12	339 (4.4)	591 (5.8)	7695	10195
	18	262 (4.0)	474 (5.9)	6559	8023
Mild/Moderate & Severe Fatigue (FAS ≥ 22)	Pre-test *	7439 (29.9)	5912 (23.4)	24866	25249
	2	4576 (31.9)	5554 (38.5)	14369	14426
	4	4202 (32.5)	5764 (37.2)	12944	15505
	6	3882 (30.6)	5149 (35.0)	12681	14693
	9	2862 (29.7)	4303 (34.8)	9631	12369
	12	2272 (29.5)	3436 (33.7)	7695	10195
	18	1846 (28.1)	2659 (33.1)	6559	8023
Post-Exertional Malaise (DSQ frequency ≥ 2 & severity ≥ 2)	Pre-test *	*Not asked*	*Not asked*	*Not asked*	*Not asked*
	2	3027 (21.1)	4174 (28.9)	14369	14426
	4	2955 (22.8)	4377 (28.2)	12944	15505
	6	2804 (22.1)	3928 (26.7)	12681	14693
	9	2113 (21.9)	3174 (25.7)	9631	12369
	12	1669 (21.7)	2569 (25.2)	7695	10195
	18	1354 (20.6)	2014 (25.1)	6559	8023

** Pre-test refers to the six-month period prior and up to the index test date*

FAS: ©Fatigue Assessment Scale, DSQ : Depaul Symptom Questionnaire

© 2024 O'Regan E et al. *JAMA Network Open.*

eFigure 3: Distribution of Fatigue Assessment Scale (FAS) scores leading up to the test and at each follow-up point, by SARS-CoV-2 test result. Pre-test refers to self-reported FAS scores in the six months leading up to the index test. Possible scores range from 10-50. Grey lines reflect cutoffs for mild/moderate fatigue (a score of 22), and severe fatigue (a score of 35). Blue vertical lines show mean scores for SARS-CoV-2 test-negatives, and red vertical dashed lines show mean scores for SARS-CoV-2 test-positives.

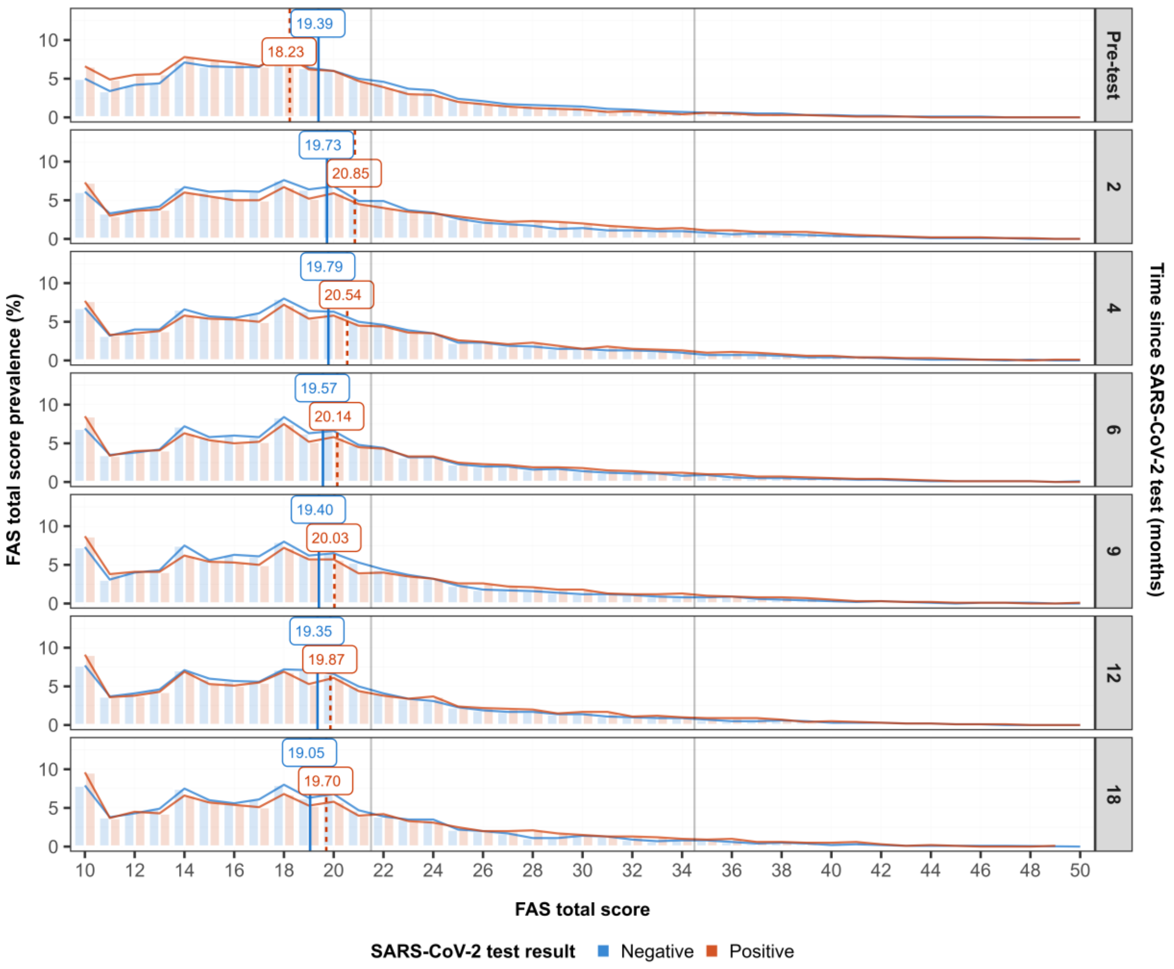

© 2024 O'Regan E et al. *JAMA Network Open.*

eFigure 4: Odds ratios (OR) and 95% confidence intervals (CI) for severe fatigue scores (scores ≥35, ref: <22) between test-positive- and negative individuals. Estimates are presented for pre-test scores*, each follow-up point, and the combined 2-18 months after testing.

Months since SARS-CoV-2 test	Negative n (%)	Positive n (%)	Positive/Negative OR (95% CI)
Pre-test	929 (3.7)	663 (2.6)	0.21 (0.17, 0.27)
2	691 (4.8)	1102 (7.6)	2.42 (1.88, 3.11)
4	602 (4.7)	1070 (6.9)	2.00 (1.54, 2.60)
6	584 (4.6)	925 (6.3)	1.76 (1.34, 2.31)
9	438 (4.6)	754 (6.1)	1.70 (1.25, 2.29)
12	339 (4.4)	591 (5.8)	1.62 (1.16, 2.26)
18	262 (4.0)	474 (5.9)	1.82 (1.26, 2.64)
2-18			1.93 (1.64, 2.26)

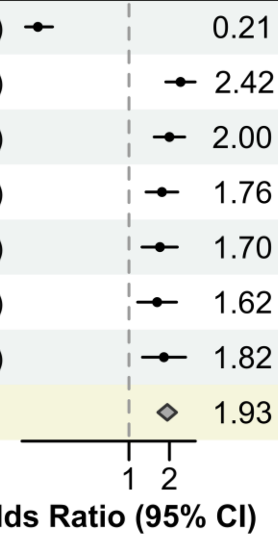

*Pre-test scores refer to scores in the six months leading up to the index test.

These logistic mixed effects models took the following fixed effects into account: age, sex, body mass index (BMI), Charlson comorbidity index (CCI), healthcare occupation, SARS-CoV-2 variant, vaccination status, employment, and education level. The individual identifier was included as a random effect.

© 2024 O'Regan E et al. *JAMA Network Open.*

eFigure 5: Odds ratios (OR) and 95% confidence intervals (CI) for substantial fatigue scores (≥22, ref: <22) between test-positive- and negative individuals. Estimates are presented for pre-test scores*, each follow-up point, and the combined 2-18 months after testing.

Months since SARS-CoV-2 test	Negative n (%)	Positive n (%)	Positive/Negative OR (95% CI)
Pre-test	7439 (29.9)	5912 (23.4)	0.48 (0.44, 0.52)
2	4576 (31.9)	5554 (38.5)	1.87 (1.69, 2.06)
4	4202 (32.5)	5764 (37.2)	1.62 (1.47, 1.78)
6	3882 (30.6)	5149 (35.0)	1.55 (1.41, 1.72)
9	2862 (29.7)	4303 (34.8)	1.56 (1.40, 1.74)
12	2272 (29.5)	3436 (33.7)	1.62 (1.44, 1.82)
18	1846 (28.1)	2659 (33.1)	1.53 (1.34, 1.74)
2-18			1.63 (1.52, 1.75)

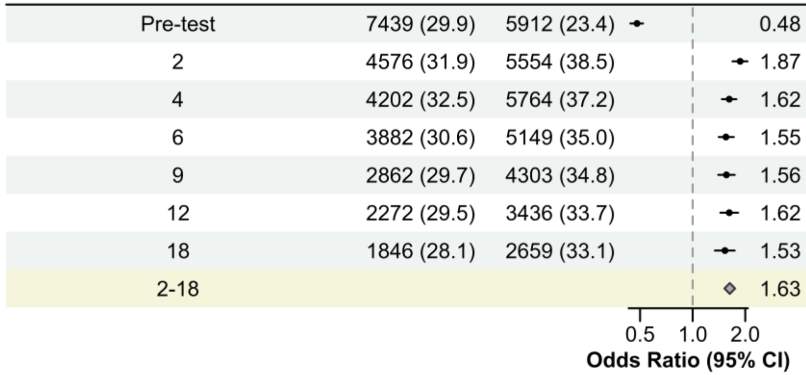

*Pre-test scores refer to scores in the six months leading up to the index test.

These logistic mixed effects models took the following fixed effects into account: age, sex, body mass index (BMI), Charlson comorbidity index (CCI), healthcare occupation, SARS-CoV-2 variant, vaccination status, employment, and education level. The individual identifier was included as a random effect.

© 2024 O'Regan E et al. *JAMA Network Open.*

eFigure 6: Score ratios (SR) and 95% confidence intervals (CI) for FAS scores 2-18 months after testing between test-positives and -negatives, stratified by possible risk factors.

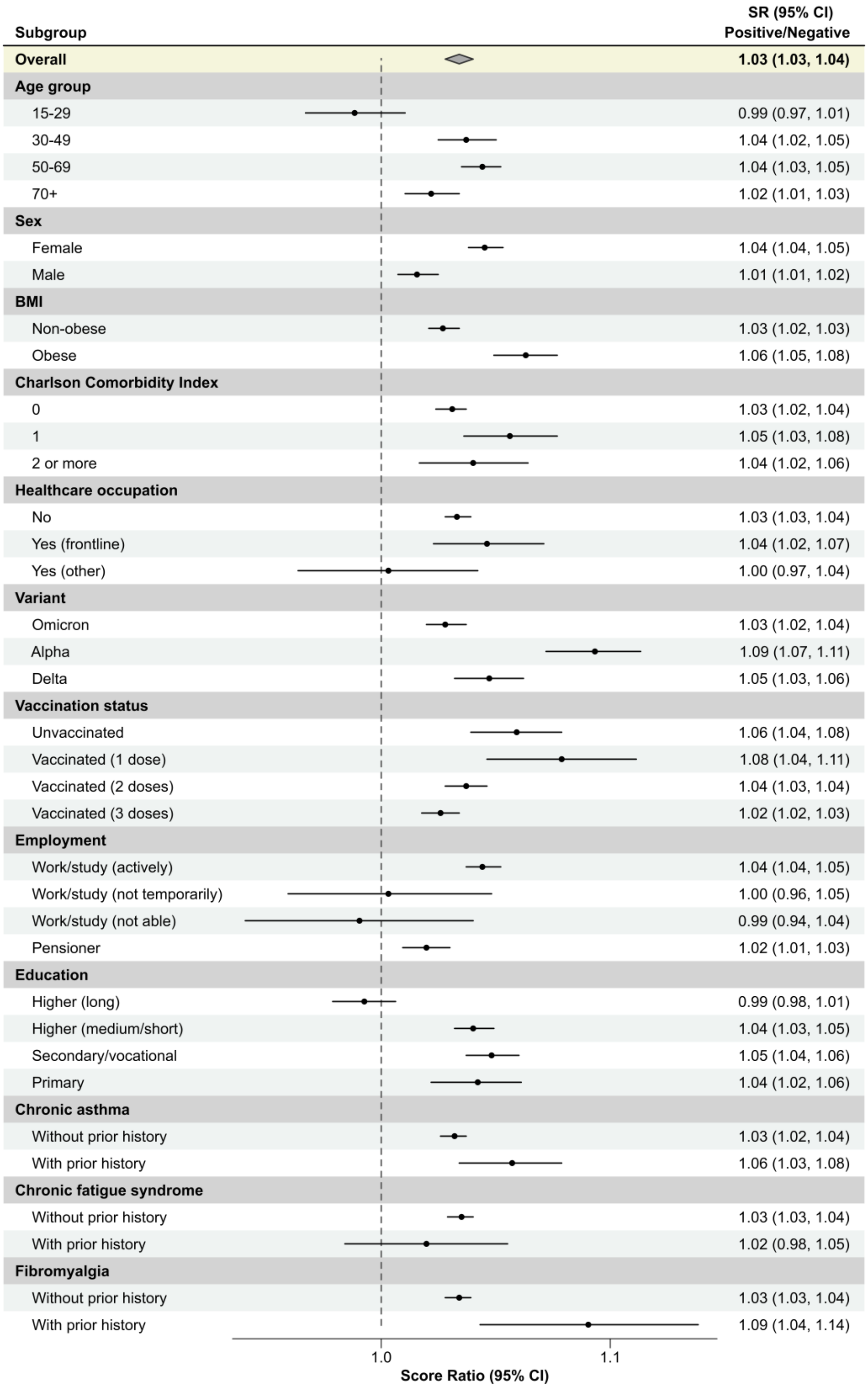

© 2024 O'Regan E et al. *JAMA Network Open.*

eFigure 7: Score ratios (SRs) and 95% confidence intervals (CI) for FAS scores across follow-up points after testing between test-positives and -negatives, stratified by dominant SARS-CoV-2 variant at time of index testing.

Months since SARS-CoV-2 test	Alpha (-) n | Mean (SD)	Alpha (+) n | Mean (SD)	Delta (-) n | Mean (SD)	Delta (+) n | Mean (SD)	Omicron (-) n | Mean (SD)	Omicron (+) n | Mean (SD)	Alpha SR (95% CI)	Delta SR (95% CI)	Omicron SR (95% CI)
Pre-test	2830 | 18.54 (6.42)	1536 | 17.91 (6.13)	4296 | 19.24 (6.50)	3002 | 17.88 (5.86)	10192 | 19.79 (6.83)	12946 | 18.54 (6.39)	0.95 (0.93, 0.97)	0.92 (0.91, 0.94)	0.94 (0.93, 0.94)
2	-	-	3203 | 19.63 (6.94)	2103 | 21.04 (8.03)	5826 | 19.95 (7.17)	7399 | 21.02 (8.00)	-	1.06 (1.04, 1.08)	1.05 (1.03, 1.06)
4	318 | 19.79 (7.25)	211 | 22.18 (8.66)	2367 | 20.00 (7.12)	1780 | 20.89 (8.13)	6716 | 19.89 (7.21)	9094 | 20.53 (7.78)	1.12 (1.07, 1.18)	1.04 (1.02, 1.06)	1.03 (1.02, 1.04)
6	2413 | 19.25 (6.73)	1288 | 21.51 (8.44)	1406 | 19.81 (7.26)	1348 | 20.60 (8.01)	5593 | 19.77 (7.24)	8026 | 20.06 (7.67)	1.10 (1.07, 1.12)	1.04 (1.02, 1.07)	1.02 (1.01, 1.03)
9	1271 | 19.37 (6.80)	660 | 20.78 (8.34)	993 | 19.23 (6.89)	1209 | 20.06 (7.71)	4486 | 19.65 (7.12)	6526 | 20.09 (7.69)	1.07 (1.04, 1.10)	1.03 (1.01, 1.06)	1.02 (1.01, 1.03)
12	640 | 18.95 (7.09)	475 | 21.08 (8.19)	803 | 18.93 (6.79)	1017 | 19.98 (7.80)	4123 | 19.74 (7.16)	5703 | 19.89 (7.56)	1.09 (1.05, 1.12)	1.05 (1.03, 1.08)	1.02 (1.01, 1.03)
18	532 | 18.95 (6.83)	398 | 21.08 (8.29)	675 | 19.21 (6.71)	841 | 20.07 (7.67)	3245 | 19.24 (6.96)	3989 | 19.69 (7.58)	1.11 (1.07, 1.15)	1.03 (1.00, 1.06)	1.02 (1.00, 1.03)
2-18							1.09 (1.07, 1.11)	1.05 (1.03, 1.06)	1.03 (1.02, 1.04)

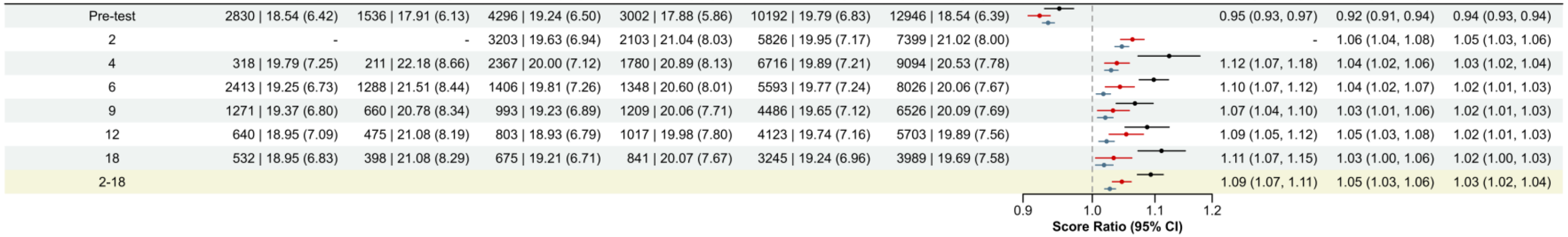

Variant — Alpha — Delta — Omicron

These Poisson mixed effects models took the following fixed effects into account: age, sex, body mass index (BMI), Charlson comorbidity index (CCI), healthcare occupation, vaccination status, employment, and education level. The individual identifier was included as a random effect.

© 2024 O'Regan E et al. *JAMA Network Open.*

eFigure 8: Score ratios (SRs) and 95% confidence intervals (CI) for FAS scores 2-18 months after testing between test-positives and -negatives, stratified by vaccination status at time of SARS-CoV-2 index test.

Months since SARS-CoV-2 test	Unvacc (-) n | Mean (SD)	Unvacc (+) n | Mean (SD)	Vacc. (1 dose) (-) n | Mean (SD)	Vacc. (1 dose) (+) n | Mean (SD)	Vacc. (2 doses) (-) n | Mean (SD)	Vacc. (2 doses) (+) n | Mean (SD)	Vacc. (3 doses) (-) n | Mean (SD)	Vacc. (3 doses) (+) n | Mean (SD)	Unvacc. SR (95% CI)	Vacc. (1 dose) SR (95% CI)	Vacc. (2 doses) SR (95% CI)	Vacc. (3 doses) SR (95% CI)
Pre-test	2534 | 18.88 (6.84)	2489 | 18.26 (6.53)	1168 | 18.99 (6.52)	640 | 18.53 (5.96)	9447 | 19.40 (6.68)	10335 | 18.17 (6.19)	11717 | 19.53 (6.61)	11785 | 18.27 (6.21)	0.94 (0.92, 0.96)	0.95 (0.92, 0.99)	0.93 (0.92, 0.94)	0.94 (0.93, 0.95)
2	332 | 21.80 (8.99)	687 | 21.59 (9.17)	383 | 19.98 (7.25)	311 | 21.92 (8.24)	6588 | 19.64 (7.02)	6493 | 20.94 (8.06)	7066 | 19.70 (6.98)	6935 | 20.65 (7.86)	1.02 (0.98, 1.05)	1.08 (1.03, 1.13)	1.06 (1.04, 1.07)	1.04 (1.03, 1.05)
4	405 | 20.56 (7.92)	780 | 21.74 (9.10)	514 | 19.92 (7.08)	355 | 21.88 (8.33)	4653 | 19.87 (7.18)	5979 | 20.85 (8.03)	7372 | 19.68 (7.05)	8391 | 20.16 (7.55)	1.06 (1.02, 1.10)	1.08 (1.04, 1.13)	1.04 (1.02, 1.05)	1.03 (1.02, 1.04)
6	1916 | 19.57 (7.03)	1635 | 21.46 (8.78)	700 | 19.06 (6.62)	284 | 21.33 (8.26)	3757 | 19.75 (7.34)	5205 | 20.19 (7.75)	6308 | 19.51 (7.02)	7569 | 19.78 (7.48)	1.07 (1.05, 1.10)	1.08 (1.03, 1.13)	1.02 (1.01, 1.03)	1.02 (1.01, 1.03)
9	972 | 19.39 (7.21)	949 | 21.12 (8.90)	362 | 19.55 (6.60)	175 | 21.03 (8.54)	3045 | 19.30 (6.98)	4853 | 20.13 (7.81)	5252 | 19.46 (6.99)	6392 | 19.76 (7.46)	1.05 (1.02, 1.08)	1.06 (1.00, 1.11)	1.03 (1.02, 1.04)	1.02 (1.01, 1.03)
12	484 | 19.12 (7.52)	697 | 20.71 (8.51)	214 | 18.34 (6.20)	150 | 21.36 (8.79)	2308 | 19.07 (7.17)	3786 | 20.04 (7.76)	4689 | 19.56 (7.04)	5562 | 19.60 (7.40)	1.06 (1.02, 1.09)	1.09 (1.03, 1.16)	1.04 (1.02, 1.05)	1.02 (1.01, 1.03)
18	402 | 19.21 (7.14)	567 | 21.02 (8.71)	191 | 18.86 (6.49)	106 | 21.69 (9.15)	2073 | 18.97 (7.02)	3332 | 19.84 (7.81)	3893 | 19.08 (6.81)	4018 | 19.35 (7.35)	1.07 (1.03, 1.11)	1.08 (1.02, 1.16)	1.02 (1.01, 1.04)	1.02 (1.00, 1.03)
2-18									1.06 (1.04, 1.08)	1.08 (1.04, 1.11)	1.04 (1.03, 1.04)	1.02 (1.02, 1.03)

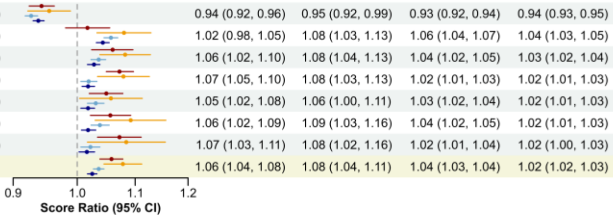

Vaccination status: Unvaccinated, Vaccinated (1 dose), Vaccinated (2 doses), Vaccinated (3 doses)

These Poisson mixed effects models took the following fixed effects into account: age, sex, body mass index (BMI), Charlson comorbidity index (CCI), healthcare occupation, dominant SARS-CoV-2 variant at time of testing, employment, and education level. The individual identifier was included as a random effect.

© 2024 O'Regan E et al. *JAMA Network Open.*

eFigure 9: Odds ratios (OR) and 95% confidence intervals (CI) for post-exertional malaise (PEM) (frequency and severity score of at least 2 and 2 on any items, REF: all other scoring) between test-positive- and negative individuals. Estimates are presented for each follow-up point and the combined 2-18 months after testing.

Months since SARS-CoV-2 test	Negative n (%)	Positive n (%)	Positive/Negative OR (95% CI)
2	3027 (21.1)	4174 (28.9)	2.92 (2.52, 3.39)
4	2955 (22.8)	4377 (28.2)	2.10 (1.81, 2.43)
6	2804 (22.1)	3928 (26.7)	1.71 (1.47, 2.00)
9	2113 (21.9)	3174 (25.7)	1.51 (1.28, 1.78)
12	1669 (21.7)	2569 (25.2)	1.71 (1.44, 2.04)
18	1354 (20.6)	2014 (25.1)	1.57 (1.31, 1.89)
2-18			2.04 (1.81, 2.30)

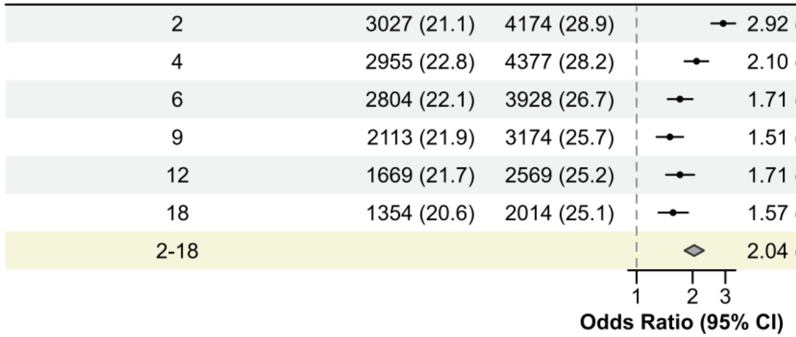

These logistic mixed effects models took the following fixed effects into account: age, sex, body mass index (BMI), Charlson comorbidity index (CCI), healthcare occupation, SARS-CoV-2 variant, vaccination status, employment, and education level. The individual identifier was included as a random effect.

© 2024 O'Regan E et al. *JAMA Network Open.*
