# Supplementary material for: Post–COVID-19 Condition Fatigue Outcomes Among Danish Residents
Source: JAMA Netw Open. 2024 Oct 7;7(10):e2434863. doi: 10.1001/jamanetworkopen.2024.34863 (PMC11581651; doi:10.1001/jamanetworkopen.2024.34863)
Supplement: Supplement 2. — Data Sharing Statement [file jamanetwopen-e2434863-s002.pdf]

Data Sharing Statement

O'Regan. Post–COVID-19 Condition Fatigue Outcomes Among Danish Residents. *JAMA Netw Open*. Published September 20, 2024. doi:10.1001/jamanetworkopen.2024.34863

Data
Data available: No

Additional Information
Explanation for why data not available: The datasets used in this study comprise sensitive, individual-level information from completed questionnaires and national register data. According to the Danish data protection legislation, the authors are not permitted to share these sensitive data directly upon request. However, the data are available for research purposes upon request to the Danish Health Authority (register data, email: kontakt@sundhedsdata.dk) and Statens Serum Institut (questionnaire data, email: aii@ssi.dk), as well as within the framework of the Danish data protection legislation and any required permission from authorities. Data request processing can take an expected 3–6 months.
